# Editorial: COVID-19 - lessons learned in pediatrics

**DOI:** 10.3389/fped.2025.1737801

**Published:** 2025-11-21

**Authors:** Michael von Rhein, Julia Dratva, Michelle Seiler

**Affiliations:** 1 Child Development Center and Pediatric Emergency Department, University Children’s Hospital Zurich, Zurich, Switzerland; 2 Institute for Public Health, ZHAW Zurich University of Applied Sciences, Winterthur, Switzerland

**Keywords:** COVID-19, lessons learned, pediatrics, health care, public health, SARS-CoV-2

The coronavirus disease 2019 (COVID-19) pandemic has profoundly impacted pediatric healthcare worldwide. It has presented unique challenges and provided essential lessons for future clinical and public health strategies in pediatrics. During the acute phase, research efforts largely focused on cross-sectional data to address immediate concerns. However, as we move beyond the crisis, it becomes increasingly crucial to reflect on the longer-term effects on children and adolescents. These include indirect impacts, lingering consequences, and vulnerabilities distinct from those of adults (1–3).

Contributions to this Research topic cover a broad spectrum—from the clinical manifestations of the disease in previously healthy children to the presentation in children with chronic conditions. Key insights addressed the understanding of pediatric long COVID symptomatology and management in youth, and the challenges of forecasting healthcare demand in uncertain times. Rather than offering definitive conclusions, these contributions invited reflection and further inquiry.

Three overarching themes emerged:

1. The Resilience and Recovery of Pediatric Health Services: Data from regions such as Northern Italy showed uneven rebounds in service utilization, emphasizing the necessity for real-time monitoring and flexible planning to address persistent gaps.
2. Heterogeneity, Stratification, and Pathobiology: COVID-19 manifests variably among children. Genetic and immunological studies suggest biomarkers for risk stratification. Qualitative research underscores the importance of integrating biological data with lived experiences to guide equitable and context-sensitive care.
3. Forecasting, Surveillance, and Anticipatory Planning: Pandemic disruptions have compromised forecasting models for pediatric respiratory infections, highlighting the need for adaptive, possibly AI-enhanced models that are resilient to systemic shocks.

Two studies focused on individual patient experiences. Kamel et al. explored long COVID in pediatric patients by describing clinical insights from a long COVID clinic. They described pediatric long COVID as marked by prolonged, multisystem symptoms, and concluded that vaccination may offer symptomatic benefits to some patients; however, larger prospective studies are necessary to better define its role (Kamel et al.). Zahnd et al. performed an exploratory qualitative study of pediatric home care, describing the health experiences of children, adolescents, and their families during the COVID-19 pandemic. The authors reported numerous challenges at the individual, familial, and community levels, and concluded that society should prepare better for the future, especially with regard to the health of children and adolescents, particularly in terms of psychosocial support for families (Zahnd et al.).

Three studies investigated healthcare utilization and models to predict health-seeking behavior. Methi and Magnusson described the accuracy of forecasts for hospital admissions due to respiratory tract infections in children aged 0–5 years for 2017 and 2023. They concluded that in scenarios similar to the COVID-19 pandemic, when the transmission of respiratory viruses is suppressed over an extended period, a simple regression model, assuming that non-hospitalized children would be hospitalized the following season, most accurately predicts hospital admission numbers. These simple forecasts may be useful for capacity planning activities in hospitals (Methi and Magnusson). Puntoni et al. described pediatric healthcare service utilization after the end of COVID-19 in a 6-year quasi-experimental study using interrupted time-series analysis. The research team found a persistent rise in hospital admissions for mental health disorders, especially among teenage girls, and concluded that data from their study and future research can inform proactive policies to mitigate disruptions and ensure access to essential pediatric services during future health crises (Puntoni et al.). This aligns with the conclusions drawn by Seiler et al., who investigated regional dynamics in pediatric emergency visits during the pandemic at three tertiary centers in Switzerland. The authors used a novel Geographic visualization technique to illustrate how the pandemic influenced pediatric healthcare utilization differently across language regions, suggesting multifactorial influences. Their conclusion was that data-based, region-specific strategies are needed to cope with future healthcare crises (Seiler et al.).

Kozak et al. introduced a different aspect of the lessons learned by conducting a pilot study on the influence of genetic polymorphisms on cytokine profiles in pediatric patients with COVID-19. They examined genes related to severe acute respiratory syndrome coronavirus 2 (SARS-CoV-2) invasion and interferon-induced immunity. Additionally, they investigated genes linked to Kawasaki disease that play a role in immunogenetics, identifying significant gene-cytokine associations in pediatric patients with COVID-19, which holds promise for the development of targeted interventions and more personalized treatment approaches (Kozak et al.).

In conclusion, the Research Topic “COVID-19 – Lessons Learned in Pediatrics” highlights the shift from short-term observations to more comprehensive, longitudinal perspectives. These contributions deepen our understanding of the full spectrum of pediatric COVID-19 disease and its aftermath, offering insights that can inform resilient care models not only for future pandemics but also for endemic respiratory diseases, such as influenza and RSV. Early in the pandemic, children were often considered relatively spared from severe acute illness. However, pediatric healthcare systems faced substantial structural challenges: preventive services were disrupted, access to care varied across regions, mental health needs surged, and uncertainties about long COVID, immunological sequelae, and virus-host interactions in developing systems remained. Notably, the majority of pandemic preparedness plans primarily targeted adults; future strategies must be tailored to the specific needs of children, adolescents, and their families. The ripple effects of the pandemic extended into pediatric health services, where routine care was disrupted and recovery proved uneven. These disruptions highlight the need for healthcare systems that are not only reactive but also resilient and capable of adapting and ensuring continuity of care for all pediatric populations. The lessons learned extend beyond emergency pandemic response into daily pediatric practice and system-wide preparedness.

To shape future pediatric pandemic readiness, four key lessons can be identified: 1.) embed longitudinal, mixed-methods designs from the onset to capture sustained effects, 2.) develop modular policies that are adaptable to diverse pediatric subgroups, 3.) ensure that resilience includes recovery and catch-up of deferred care, and 4.) build robust forecasting frameworks that recalibrate dynamically under disruption.

To translate these insights into practice, pediatric cohorts should be maintained during interepidemic periods, surveillance should be integrated across all pathogens, adaptive recovery protocols should be implemented, and equity in pediatric access should be assured through data-driven spatial and social analyses.

This Research Topic serves as both a reflection and a foundation for stronger, more resilient pediatric healthcare systems that are prepared to face future challenges. We thank all contributing authors for their rigorous work. May this collection serve not only as a reflection on the past, but also as a seedbed of better preparedness for future challenges to child health.
